# Error Recovery in the Time-Triggered Paradigm with FTT-CAN

**DOI:** 10.3390/s18010188

**Published:** 2018-01-11

**Authors:** Luis Marques, Verónica Vasconcelos, Paulo Pedreiras, Luís Almeida

**Affiliations:** 1 Instituto Superior de Engenharia de Coimbra, Rua Pedro Nunes, 3030-188 Coimbra, Portugal; veronica@isec.pt; 2 Instituto de Telecomunicações, Dep. Electrónica, Telecomunicações e Informática, Universidade de Aveiro, 3810-193 Aveiro, Portugal; pbrp@ua.pt; 3 Instituto de Telecomunicações, Faculdade de Engenharia, Universidade do Porto, 4200-465 Porto, Portugal; lda@fe.up.pt

**Keywords:** flexible time-triggered, fault-tolerance, time-triggered, CAN, server, scheduling, temporal redundancy, real-time systems

## Abstract

Data networks are naturally prone to interferences that can corrupt messages, leading to performance degradation or even to critical failure of the corresponding distributed system. To improve resilience of critical systems, time-triggered networks are frequently used, based on communication schedules defined at design-time. These networks offer prompt error detection, but slow error recovery that can only be compensated with bandwidth overprovisioning. On the contrary, the Flexible Time-Triggered (FTT) paradigm uses online traffic scheduling, which enables a compromise between error detection and recovery that can achieve timely recovery with a fraction of the needed bandwidth. This article presents a new method to recover transmission errors in a time-triggered Controller Area Network (CAN) network, based on the Flexible Time-Triggered paradigm, namely FTT-CAN. The method is based on using a server (traffic shaper) to regulate the retransmission of corrupted or omitted messages. We show how to design the server to simultaneously: (1) meet a predefined reliability goal, when considering worst case error recovery scenarios bounded probabilistically by a Poisson process that models the fault arrival rate; and, (2) limit the direct and indirect interference in the message set, preserving overall system schedulability. Extensive simulations with multiple scenarios, based on practical and randomly generated systems, show a reduction of two orders of magnitude in the average bandwidth taken by the proposed error recovery mechanism, when compared with traditional approaches available in the literature based on adding extra pre-defined transmission slots.

## 1. Introduction

Today, a myriad of systems that are used directly or indirectly in our daily lives, e.g., cars, planes, and medical equipment, are controlled by distributed computing systems composed of sets of nodes that communicate with each other using communication networks to fulfill their global objectives. One network technology that is widely used to support short data exchanges is the Controller Area Network (CAN) [1], with more than 700 million controllers sold every year [2]. In the particular case of the automotive domain, CAN continues being the dominant network, despite the appearance of other contending technologies, e.g., FlexRay [3], Ethernet [4], and Real-Time Ethernet variants (e.g., AVB [5] and TSN [6]), or even the enhanced version of CAN with higher bandwidth, CAN-FD [7]. Thus, we believe the work presented in this paper, based on CAN, but that can also be directly applicable to CAN-FD with minor changes, is still relevant in practice.

Some of the subsystems where CAN is used, e.g., active safety mechanisms, exhibit high safety requirements, and can benefit from using a time-triggered (TT) design approach. In TT systems, all activities, including message transmissions, are triggered in precise time instants to avoid contention in the access to shared resources, notably the bus. This approach has been typically implemented in a static way, where the messages’ schedule is obtained offline and saved in a table with the triggering instants [8]. Naturally, this implementation approach does not tolerate the inclusion or removal of messages at run-time that were not defined in the design phase. This has limited the applicability of the TT approach to cases in which applications are either static or have a predefined set of operational modes that can then be switched online. Moreover, operational modes are not suitable to deal with errors.

Concerning transient errors, message recovery can be accomplished using temporal redundancy, spatial redundancy, or both. Spatial redundancy implies physical replication of nodes and/or transmission media, so messages are transmitted using different paths. In this case, the implementation costs can rise significantly and a more elaborated management is needed. Nevertheless, if the recovery of permanent errors is mandatory, then spatial redundancy must be used. Transient errors, which are a couple of orders of magnitude more frequent than permanent ones, can be mitigated with temporal redundancy, i.e., sending message replicas in different time instants, using the same communication path. In this paper, we consider time redundancy mechanisms, only, which are suitable to cases in which spatial redundancy is too costly or when permanent and transient faults must be simultaneously tolerated.

Time redundancy can be implemented in a systematic way or only when errors are detected. The former approach is typical in static TT approaches such as in the static segment of FlexRay [3]. In this case, error detection leads to simply discarding the corrupted message and signaling the application. In this approach, the recovery is typically done with the following instance of the affected message. Other protocols use automatic retransmission by the sender upon error detection, e.g., CAN [1]. This approach typically allows for a faster recovery and does not allocate unnecessary bandwidth in the absence of errors. However, a proper mechanism to bound the (unscheduled) retransmissions is needed, e.g., retransmissions within slots (see Section 2), or else the communication system timeliness and predictability can be compromised. Some earlier approaches to assess the impact of errors on the timeliness of CAN, using its native event-triggered (ET) model [9,10], bounded stochastically the retransmissions load using an error model. Naturally, the analyses they proposed is tightly coupled with the accuracy of the error model and, if errors beyond the model happen, the timeliness of the whole system is compromised. This differs from our approach, in which the overall system timeliness is always guaranteed, and error model violations only have impact on the probability of transient error recovery. For this reason, we will not further consider such ET approaches.

In this paper, we propose designing TT systems with a dynamic approach based on the Flexible Time-Triggered (FTT) paradigm, which is a communication paradigm that combines TT communications with online scheduling. This feature allows for scheduling message transmissions in response to particular events, such as transmission errors, thus reducing the error recovery time and bandwidth utilization. Moreover, as message retransmissions are explicitly scheduled, it becomes possible to control the impact of such events on the remaining traffic and so maintaining the determinism of the TT approach. In this work, we consider the FTT implementation over CAN (FTT-CAN protocol). However, the recovery mechanism herein presented can be applied to other broadcast-based communication media that support the FTT paradigm, such as FlexRay [11]. The application to CAN-FD is also immediate.

In previous work [12], we considered a restricted fault model with a maximum of one fault per Elementary Cycle (EC). This paper removes this constraint, allowing for a more generic and realistic fault model, where faults are directly modeled by a Poisson process. Moreover, the paper also proposes a new mechanism that supports concurrent scheduling of multiple consecutive retransmissions. This mechanism is unique and essential to obtain high delivery probability for messages with short deadlines. The new error and recovery scenarios are identified and thoroughly described and analyzed. A method for computing a probabilistic guarantee for timely message transmission upon errors is also provided. Finally, the paper also presents a method to compute the amount of resources that must be allocated to the time-triggered traffic to guarantee that all the periodic messages in the system are correctly delivered to their recipients within a given reliability target.

The paper is organized as follows: Section 2 presents the related work concerning temporal redundancy mechanisms in TT networks and Section 3 describes the FTT-CAN protocol and its underpinnings, detailing also the new fault model. Section 4 presents the error-recovery proposal, including an analysis of the error and recovery scenarios. In Section 5, the methods and algorithms necessary to minimize the used bandwidth are presented, followed by Section 6, where the proposal is assessed. Section 7 describes the updates to the FTT-CAN protocol that must be implemented to support the functionality of the error recovery mechanism. Finally, Section 8 concludes the paper. Appendix A lists all acronyms and B the used benchmark message sets.

## 2. Background and Related Work

Time-Triggered protocols, e.g., TTP/C [13], have traditionally used a Time-Division Multiple Access (TDMA) medium access mechanism, in which time is divided in slots that are exclusively and statically allocated to nodes, and within which predetermined messages are transmitted. This bandwidth allocation method grants these systems a predictable and steady behavior under any operational scenario, which is an important characteristic for the certification of safety-critical systems, e.g., X-by-wire systems [14]. On the other hand, this method is rather inflexible in what concerns adjusting to varying operational conditions, either due to system reconfigurations or rare events. For instance, if an alarm generates a sporadic message with a deadline equal to 5 ms and an average inter-arrival time of 1000 ms, a static TDMA-based TT system must reserve a slot every 5 ms to guarantee a timely response to the alarm. Most of the time the slot is not used, contributing this way to a low bandwidth efficiency. The same happens in what concerns error recovery, since errors can occur at any time instant and require a reaction in an interval that is typically much shorter than average error inter-arrival time. Therefore, static TDMA systems that need high reliability require the transmission of several message instances that most of the times are useless.

In FlexRay networks the TT traffic is allocated statically to slots in the Static Segment (SS). Tanasa et al. [15] propose a method to recover from message errors in the SS that basically defines the number of copies of each message that must be sent to obtain a global success probability (*GP*) that must be greater than the intended system reliability, *ρ*, for a given mission time. This can be achieved using Equation (1), where *N* is the number of messages, *p_i_* the error probability of message *i*, obtained from the Bit Error Rate (BER) and number of transmitted bits, MT is the mission time, T_i_ the period of message *i,* and *k_i_* is the number of extra copies of message *i* that shall be sent.

(1) $$GP=\overset{N}{\underset{i=1}{\prod }}{\left(1-p_{i}^{k_{i}+1}\right)}^{\frac{MT}{T_{i}}}>\rho$$

After obtaining all the *k_i_* values, a method based on Mixed-Integer Linear Programming (MILP) is used to minimize the number of static slots used for message replicas. The results presented there point to at least the duplication of the necessary bandwidth for typical BER values. 

The work in [16] presents the COSMIC middleware applied to a time-triggered CAN network, implementing a TDMA access scheme. It shows a method to recover errors in real-time messages. These messages are sent in dedicated offline-scheduled slots that are enlarged to allow for retransmissions using the CAN native error recovery method. The slot enlargement is then dependent on the number of faults foreseen by the fault model. This method is still inflexible, since the slots are scheduled at pre-runtime. Nevertheless, at runtime, the bandwidth assigned to retransmissions but not effectively used can be reclaimed to carry sporadic and non-real-time traffic, to allow for a more efficient bandwidth utilization. This is done simply by assigning higher CAN IDs (lower priority) to event-triggered or sporadic messages. 

Short et al. [17] present a mechanism to guarantee the message transmission in TDMA-based CAN networks. Messages are transmitted in specific windows or slots, which are enlarged to make room for CAN’s native automatic message retransmissions upon errors, but only if the retransmissions fit in the defined window. The paper presents a method to calculate the window size together with a simulation study that points to a bandwidth utilization reduction between 3% and 30%, on average, depending on the environment type (from Benign/Normal to Aggressive/Hostile, respectively) [18], when compared with the case where a predefined number of message copies is sent to attain the same reliability level. The paper includes a test case where the mechanism is applied to a critical message with 8 bytes payload, a period of 100 ms and an intended transmission reliability of 10^−9^ errors per hour and using an Aggressive environment with BER equal to 2.6 × 10^−7^, showing that using the windowed method the intended reliability level is obtained with a window 26.4% shorter than using multiple copies, which in this case, implies the transmission of four copies. The implementation of this mechanism implies building specific hardware (FPGA based) for the nodes and is still inflexible in what concerns modifying the message set dynamically, as the schedule is built offline.

In the scope of FTT-CAN, slack time can be placed in the synchronous window to allow the automatic retransmission of messages affected by errors [19]. This approach is more bandwidth efficient than the previous ones, since the extra-time allocated in the synchronous window is shared by all the TT messages sent in the current EC. The amount of slack time is dependent on the maximum number of errors considered in each EC and is always wasted when there are no errors, thus limiting the efficiency of the approach. Nevertheless, this approach allows for the recovery in the same cycle where errors occurred.

In this paper, we use a novel approach, which is based on a server that allocates dynamically bandwidth for retransmissions. The server parameterization uses a Poisson-based fault-model, allowing to guarantee a timely recovery of errors up to a given desired probability target. As opposed to previous works, in our proposal bandwidth is consumed only when errors do actually happen and the interference in the remaining TT traffic is strictly bounded by the server parameters, thus boosting the bandwidth efficiency.

## 3. System Model

This section presents a global overview of the system model considered in this work, including a short review of the FTT-CAN protocol, associated schedulability analysis, scheduling servers, and fault model.

### 3.1. The FTT-CAN Protocol

**Figure 1:**
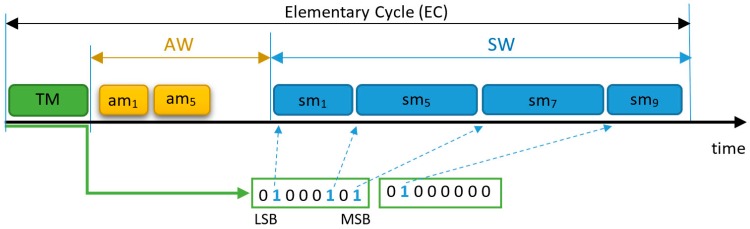
Elementary Cycle (EC) and Trigger Message (TM) encoding in Flexible Time-Triggered -Controller Area Network (FTT-CAN).

According to the FTT paradigm [20], the network time is divided in a succession of ECs (Figure 1), with a preconfigured fixed duration, which constitutes the temporal resolution of the traffic. FTT-CAN is a Master-Slave protocol and the master node schedules the TT traffic online, for each EC, communicating the schedule to the slave nodes using a Trigger Message (TM) transmitted at the beginning of each EC. FTT-CAN also supports ET messages, which are triggered autonomously by each node. The EC is composed of two windows, designated Asynchronous Window (AW) and Synchronous Window (SW), which carry the ET and TT traffic (respectively). The duration of each SW depends on the TT traffic scheduled for that EC (Figure 1) and is communicated to the nodes in the respective TM. The FTT paradigm was already implemented and demonstrated using several underlying technologies, such as CAN [20], Ethernet [21], and switched Ethernet [22,23]. 

The FTT-CAN protocol implementation uses a simplex bus and the TM encodes in its payload the messages to be transmitted in that EC using one bit per message request (Figure 1). Each slave decodes the TM, and, at the beginning of the SW, triggers the transmission of scheduled messages, for which it is the producer. FTT-CAN only controls which messages are transmitted within the SW, not defining a particular order, which tends to follow the native CAN arbitration scheme, with possible priority inversions due to practical technological issues, like the node’s latency. 

The Master uses an online scheduler that can implement any scheduling policy, e.g., FP (Fixed Priority), RM (Rate Monotonic), or EDF (Earliest Deadline First), being independent of the arbitration process of the underlying network technology. This node possesses a database, the System Requirements Database (SRDB), with the attributes of the messages and other system operational parameters, e.g., EC duration. The message set can be updated online using special control messages, e.g., to add and remove messages or modify their attributes. All such requests are directed to the Master node and subject to an admission control mechanism, being accepted only if they result in feasible system configurations. Event messages are triggered autonomously by the end-nodes, relying on the native CAN arbitration mechanism to prioritize and serialize concurrent transmissions. End-nodes are responsible for confining the event traffic to the AW. To do so, they use the information contained on the TM to determine the AW duration and suspend transmission at the appropriate times. A more in depth explanation of TT and ET traffic scheduling on FTT-CAN can be found in [20].

The FTT-CAN protocol has some critical points, which could hinder its utilization in systems where high levels of reliability and dependability are mandatory. For instance, the Master node is essential to the correct operation of an FTT-CAN network, constituting a single-point of failure. The work in [24] addresses the Master node replication to cope with permanent failures. Also, a solution to guarantee the TM delivery when this message is hit by errors or the Master suffers a transient fault is presented in [19]. To deal with bus permanent failures a bus replication scheme is proposed in [25], which could also increment the available bandwidth and/or increase system dependability.

### 3.2. Schedulability Tests in FTT-CAN and Real-Time Performance

**Figure 2:**
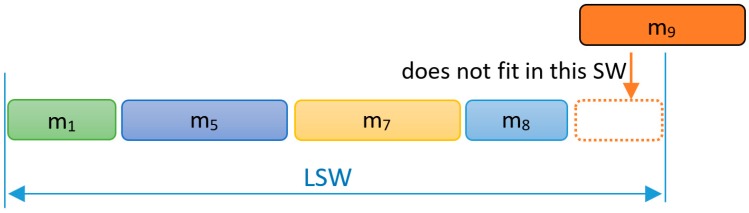
Insert idle time in the Synchronous Window (SW).

In FTT-CAN, when scheduling the synchronous messages for the next EC, some idle time may appear at the end of the SW (Figure 2). This Inserted Idle Time (IIT) is essential to allow for the transmission of the TM without any blocking. The actual amount of IIT added in each EC depends on the traffic scheduled for that SW and it can be upper bounded by the length of the longest ready message whose transmission must be postponed to a future EC to avoid a TM overrun, as would be the case of message m_9_ in Figure 2. The upper bound of the IIT is denoted by *X* in Equation (2). The scheduling model with IIT that is used in FTT-CAN is the Blocking-Free Non-Preemptive scheduling model [26]. Traffic schedulability in this model can be assessed as if the scheduling was fully preemptive, e.g., using common response time analysis (RTA) [27], as long as the message transmission times are inflated as in Equation (2), where LEC represents the EC duration and LSW the maximum SW duration.

(2) $$C_{i}^{E}=\frac{LEC}{LSW-X}C_{i}$$

Therefore, when considering an FTT-CAN system with a message set *M* as in Equation (3) with *n* messages characterized by a maximum transmission time *C_i_* (including maximum bit-stuffing), a period *T_i_*, and deadline *D_i_* ≤ *T_i_*, schedulability can be guaranteed if an upper bound to message *i* response time (R_i_) when considering the inflated transmission time ($C_{i}^{E}$), as in Equation (4), is lower than or equal to the respective deadline (*D_i_*) for all *n* messages. Equation (4) can be solved with a common fixed-point iteration method and *hpe*(*i*) stands for the set of messages having higher or equal priority than message *m_i_*.

(3) $$M=\left\{m_{i}\left(C_{i},T_{i},D_{i}\right),i=1\ldots n\right\}$$

(4) $$R_{i}=C_{i}^{E}+\underset{k\in hpe\left(i\right)}{\sum }\lceil \frac{R_{i}}{T_{k}}\rceil C_{k}^{E}$$

### 3.3. Servers for Aperiodic Messages

Servers are software entities that act as proxies for associated aperiodic requests, shaping their arrival pattern and allowing for their integration in periodic/sporadic systems. Many server types can be found in the literature [28], being typically characterized by a certain capacity *C_S_* that can be provided over a given interval *T_S_* to serve arriving requests. However, they differ in the rules on how and when their capacity can be used and replenished. A Deferrable Server (DS) [29,30] replenishes its capacity strictly periodically and allows for consuming its remaining capacity at any point of its period. The server is marked as ready and scheduled whenever it has pending requests to serve and has enough capacity. The capacity is decremented by the exact amount of requested execution time that was actually served. Despite presenting a penalization in terms of the schedulability of lower-priority periodic messages, when compared to other servers, like Polling and Sporadic Servers, the simplicity, small overhead, and responsiveness of DSs make them a good practical option as referred in [31], and so they will be used in this work to handle retransmission requests.

### 3.4. Fault Model

Communication systems are subject to perturbations caused by multiple sources, such as Electro-Magnetic Interference, high energy particles, and loose connectors. From the system point of view, these perturbations can induce faults that can lead to message transmission errors.

In this work, we consider that fault arrivals follow a Poisson process (as in [10]), which possesses a random exponential time between arrivals with an average of λ faults per second (λ is also referred as process intensity). Equation (5) shows the probability of having *k* faults in a time interval τ, as a function of λ, *k* and τ. According to the Poisson process definition, this probability is independent of past events.

(5) $$P_{\lambda }\left(k;\tau \right)=e^{-\lambda \tau }\frac{{\left(\lambda \tau \right)}^{k}}{k!}$$

The average fault arrival rate λ can be obtained from experimental data available in the literature. We will rely on the numbers presented in [18], which used an experimental setup to measure the BER in three types of operational environments, denoted Benign, Normal, and Aggressive. For instance, a factory plant with several welding machines near the nodes was used as representative of an Aggressive environment. These experiences were conducted using a CAN bit-rate of 1 Mbps, and, for reference and future use, the measured values are the ones presented in Table 1.

Knowing the bit-rate of the CAN network, the length of the elementary cycle, the length of the synchronous window, and the type of environment where the system is deployed, Equation (5) can be used to compute the probability of error occurrence. These values will be used in the remainder of this paper to compute diverse parameters of the error recovery mechanisms.

In the following, we assume that the automatic retransmission of messages by the CAN controllers is disabled, to avoid jeopardizing the schedule defined in the TM, and that the Master can detect, in a consistent way, the errors occurring in the communication channel. Potential inconsistent error detection is addressed in Section 7. Bus partitions are not considered. The fault model is basically the same as the one presented in [12] concerning the arrival of faults, but without the restriction of a maximum of one fault per EC, resulting on a more realistic scenario. 

## 4. Error Recovery in FTT-CAN

Unlike the methods reviewed in Section 2, in FTT-CAN message retransmissions are scheduled dynamically, in a controlled way, in response to actual errors [12]. This section describes how the error detection and rescheduling mechanisms are implemented on FTT-CAN, and presents an analytic formulation for computing the number of replicas required to attain a desired reliability level, applicable to messages that have short deadlines.

### 4.1. Error Detection and Retransmission Scheduling Mechanisms

**Figure 3:**
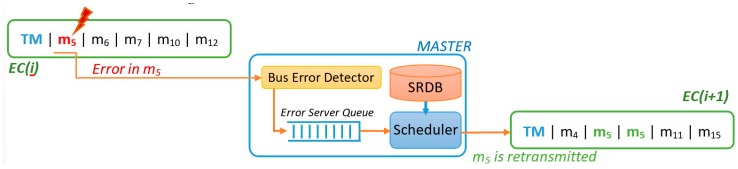
Error recovery process.

In FTT-CAN networks, the Master node is responsible for all the scheduling decisions concerning the TT traffic. Moreover, CAN is a broadcast bus, thus all of the nodes are potential listeners of all messages. The combination of these two characteristics enables the deployment of a simple but effective error detection mechanism. The Master is configured in promiscuous mode and, after sending the TM, listens to the bus, identifying the messages actually transmitted, as shown in Figure 3. 

The “Bus Error Detector” block compares the list of scheduled messages with the messages received in that EC, thus identifying eventual errors and omissions. The IDs of such messages are then put in the “Error Server Queue”. At the end of the EC the Master schedules the traffic for the next EC, when considering both the active TT messages and the messages affected by errors, contained in the “Error Server Queue”, as shown in Figure 3. Any server policy can be used to manage the error queue, provided that it is compatible with the traffic scheduler. In the remainder of this section, we assume that the server has maximum priority, to maximize responsiveness to errors, and that it has enough capacity to accommodate all the retransmission requests with a desired probability. Server design is addressed in the end of the section.

In FTT-CAN, message’s periods and deadlines are expressed as integer multiples of LEC. As such, LEC is obtained by calculating the Greatest Common Divisor of these message attributes. However, as the rescheduling mechanism herein proposed implies that message retransmissions are possible, at best, in the EC that follows the error, LEC has to be halved when it matches the period or deadline of the fastest(s) message(s).

### 4.2. The Need for Multiple Replicas

**Figure 4:**
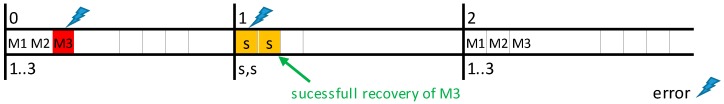
Message and replica hit by errors.

Errors may affect equally messages and their replicas and may occur more than once per EC. Therefore, assuring (probabilistically) the delivery of messages with short deadlines may require re-scheduling several replicas for the same EC, as illustrated in Figure 4, where S stands for the error-server execution (notice that the error recovery would fail if single replica was used by the server).

As fault arrivals are modeled by a Poisson process, it is not possible to upper bound the number of faults in any given time interval. However, for a fixed time interval, as the number of faults increases, their probability of occurrence decreases. Therefore, it is possible to compute the number of replicas that guarantees successful and timely transmissions with a reliability goal *ρ* > 1 − *ε_G_*, where *ε_G_* is the maximum probability of failure or system/global unreliability level.

The system reliability level is specified in acceptable errors per mission, where a generally accepted mission time is one hour [15], thus leading to a common metric of acceptable errors per hour. The system unreliability objective *ε_G_* can be converted to the error probability that each individual message may tolerate, when considering the messages periods and mission time, being this probability named *pε_i_*. Equation (1) can be used and a good approximation of it is obtained using the first two terms of the Taylor series expansion, possible due to very small values of *pε_i_*. The new formulation can be solved by upper bounding the *i* terms, using the smallest value of *T_i_* and the biggest *pε_i_* (worst GP value will be obtained). Afterwards making all *n* elements equal to this bound and applying again Taylor series approximation, as previously, the limit probability *pε_i_* can be obtained. These steps are depicted in Equations (6) and (7).

(6) $$GP=\overset{n}{\underset{i=1}{\prod }}{\left(1-p\varepsilon_{i}\right)}^{\frac{MT}{T_{i}\times LEC}}≅\overset{n}{\underset{i=1}{\prod }}\left(1-\frac{MT}{T_{i}\times LEC}p\varepsilon_{i}\right)≅1-n\times \frac{MT}{T_{i}\times LEC}p\varepsilon_{i}$$

(7) $$1-n\times \frac{MT}{T_{i}\times LEC}p\varepsilon_{i}=1-\varepsilon_{G}\Leftrightarrow p\varepsilon_{i}=\frac{\frac{\varepsilon_{G}}{\left(\frac{MT}{T_{i}\times LEC}\right)}}{n}$$

Equation (7) then defines an acceptable fail probability for a given message *i*, where *T_i_* is the message period (in number of ECs), MT is the mission time and *n* is the number of messages subject to the recovery mechanism. This equation shows that the most demanding messages, i.e., with lower *pε_i_*, are those with smallest period. Since the error-handling mechanism is the same for all messages, design decisions will be made while considering the smallest *pε_i_* value, denoted as *pε*. 

**Figure 5:**
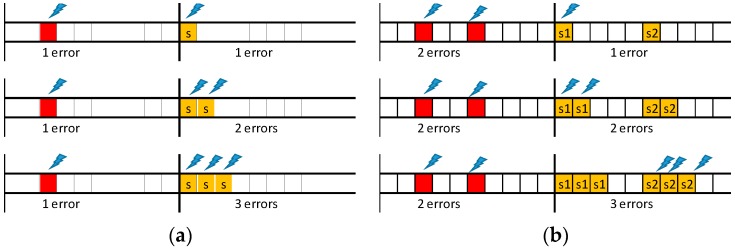
Scenarios with 1 error (**a**) and with 2 errors (**b**) and retransmission with 1 to 3 replicas.

Figure 5 depicts a set of representative error scenarios that will be used to obtain a general expression for computing the number of replicas needed to attain a given system reliability level. In practice, the values of λ, *LSW* and *pε* limit the number of scenarios that must be considered.

Let us start with the “one error/one replica” scenario, shown on the top timeline of Figure 5a. This event sequence occurs if one error affects a TT message, event with probability *P_λ_*(1; LSW), and the corresponding replica is also affected by an error, event with probability *P_λ_*(1; C*_i_*). As errors are independent, the resulting probability (*p_1/1_*) is given by the product of both probabilities. Therefore, the probability of this scenario is given by Equation (8), where message transmission times are upper bounded by *C_MAX_*, to enable the derivation of generic equations. If the probability obtained via Equation (8) is lower than *pε*, then a single replica is enough to guarantee the desired message transmission reliability level. Otherwise, an additional replica must be sent. This scenario is shown in the second timeline of Figure 5a. This case is a simple extension of the previous one, in which we consider the combined probability of both replicas being hit by faults. The probability of occurrence of this scenario (*p_1/2_*) is given by Equation (9). It is immediate to see that the probability for the scenario “one error/n replicas” (*p_1/n_replicas_*), is given by Equation (10). The smallest number of replicas that makes Equation (10) lower than *pε* is sufficient to attain the desired global reliability level for this scenario.

(8) $$p_{1/1}=P_{\lambda }\left(1;\;LSW\right)\times P_{\lambda }\left(1;\;C_{MAX}\right)$$

(9) $$p_{1/2}=P_{\lambda }\left(1;\;LSW\right)\times P_{\lambda }{\left(1;\;C_{MAX}\right)}^{2}$$

(10) $$p_{1/n\_replicas}=P_{\lambda }\left(1;\;LSW\right)\times P_{\lambda }{\left(1;\;C_{MAX}\right)}^{n\_replicas}$$

Since the error model allows for the occurrence of multiple errors in one EC we will now consider the scenarios in which two TT messages scheduled for the same EC are affected by errors (Figure 5b). When considering first that a single replica per detected error is sent, if an error hits one of the replicas the recovery process fails. The probability of failure of the recovery process (*p_2/1_*) is then simply obtained by adding the probabilities of both these combinations, each one with probability *P_λ_*(2; *LSW*), probability of having two errors in the SW, times *P_λ_*(1; *C_MAX_*), probability of one replica error, as expressed in Equation (11). Note that the scenario where both replicas are hit is also possible, but with much lower probability, so this contribution is not included in the equation. When considering now that two replicas per message are sent, a failure of the recovery process occurs only if both replicas of the same message are affected by errors, event with probability *p_2/2_*, expressed in Equation (12). Iterating the reasoning it is possible to obtain Equation (13), which allows for computing the probability (*p_2/n_replicas_*) of non-recovery for the “two errors/n replicas” scenario.

(11) $$p_{2/1}=2*P_{\lambda }\left(2;\;LSW\right)\times P_{\lambda }\left(1;\;C_{MAX}\right)$$

(12) $$p_{2/2}=2*P_{\lambda }\left(2;\;LSW\right)\times P_{\lambda }{\left(1;\;C_{MAX}\right)}^{2}$$

(13) $$p_{2/n\_replicas}=2*P_{\lambda }\left(2;\;LSW\right)\times P_{\lambda }{\left(1;\;C_{MAX}\right)}^{n\_replicas}$$

The same methodology can be applied to obtain the generic expression for scenarios with arbitrary number of errors and replicas, stated by Equation (14).

(14) $$p_{n\_errors/n\_replicas}=n\_errors\times P_{\lambda }\left(n\_errors;\;LSW\right)\times P_{\lambda }{\left(1;\;C_{MAX}\right)}^{n\_replicas}$$

### 4.3. Server Capacity Computation

Message retransmissions require bus time, thus having impact on the response time of the remaining messages. Due to the unpredictable nature of errors’ occurrence, it is necessary to bound such interference, at least when message timeliness guarantees are a system requirement. We decided to use a server to manage the retransmissions because, in addition to this functionality, a server:

- Is resource-efficient, since it consumes bandwidth only when activated, i.e., in the presence of errors;
- allows controlling the reactivity to errors via its associated priority and budget/period;
- is predictable and analyzable.

The analysis that follows allows computing the server capacity, extending the one presented in [12], by taking into account the need for multiple retransmissions to achieve a desired reliability level, as discussed previously. 

**Figure 6:**
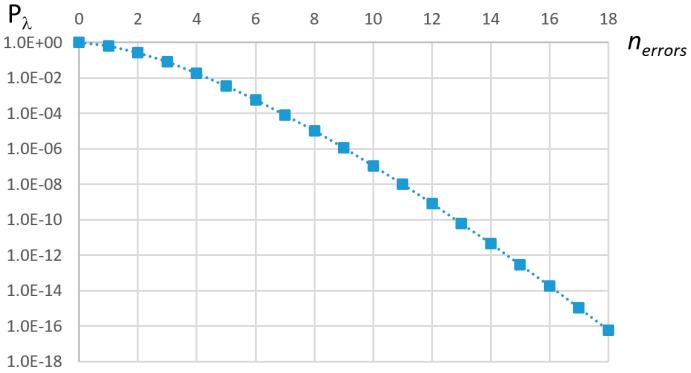
Plot of *P_λ_* (≥ *n_errors_*; 1/λ)

Equation (5) allows for computing the probability of having *k* faults over any given time interval τ. It is however useful to use as system design metric the probability of non-recovery, in the server period, designated *pε_S_*, which can be computed with Equation (15) for a Poisson process. *P_λ_* (≥n; τ) is plotted in Figure 6 with τ = 1/λ and an Agressive environment. Based on this equation, it is possible to determine the number of errors that must be accommodated by the error-handling mechanism, and, from this, compute the minimum server capacity for its specified period *T_S_*.

(15) $$P_{\lambda }\left(\geq n_{errors};\;T_{S}\right)<p\varepsilon_{S}$$

For example, considering one server period (*T_S_* = τ), τ = 1/λ and a target of *pε_S_* = 10^−10^, the minimum value of *n_errors_* that satisfies Equation (15) is 13 (see Figure 6). *T_S_* was chosen heuristically, to match a mean expected number of errors of one. It was shown in [12] that the server bandwidth increases as the server period decreases, so there is no advantage in attributing smaller values to *T_S_*. Noting that it is not possible to foresee which messages will be affected by errors, the worst-case situation happens when all of them must be transmitted in the following EC, implying scheduling several replicas at once, as discussed previously. Assuming *z* replicas per error, the server must then have a capacity equal to 13 × *z* maximum length messages. For instance, if the maximum replica level is equal to 3 (i.e., 3 replicas allow attaining the desired reliability) and LEC is equal to 2.5 ms, then the recovery mechanism uses 0.14% of the system bandwidth (*C_MAX_* = 135 μs and *T_S_* = 1/λ = 3.85 s), in the worst case, which represents a negligible fraction of the available bandwidth. Nevertheless, as we will see in the following section, the interference caused by the server execution on the scheduling of the remaining messages is non-negligible, and must be accounted for in the schedulability analysis.

## 5. Resource Optimization Process

This section presents methods to compute the worst-case response time (WCRT) of messages, taking into consideration the interference of the server and an algorithm for minimizing the size of the synchronous window, complementing the analysis presented in the previous section.

### 5.1. Server Interference

To obtain the WCRT of all messages we need to extend Equation (4), to consider the server interference, represented by the intermediate term (the summation of *Interf_P*) and the error signaling (the summation of *Err_S*) in Equation (16). The first term depends on the errors that occurred in the previous SW and on the replication level (as explained at the end of the next section) and the second one depends on the errors in the current SW. The response time must be calculated for each error scenario and server interference, accounted by the *l* variable.

(16) $$R_{i}^{n+1}\left(l\right)=C_{i}^{E}+\sum_{j=1}^{EC_{number}\left(R_{i}^{n}\left(l\right)\right)}\left(Interf\_P\left(l,\;j\right)\times C_{MAX}^{E}+Err\_S\left(l,\;j\right)\times C_{error}^{E}\right)+\sum_{k=1}^{i-1}\lceil \frac{R_{i}^{n}\left(l\right)}{T_{k}}\rceil C_{k}^{E}$$

The $EC_{number}\left(R_{i}^{n}\left(l\right)\right)$ variable, obtained with Equation (17), is the number of ECs that need to be analyzed in iteration *n* for message *i*.

(17) $$EC_{number}\left(R_{i}^{n}\left(l\right)\right)=\lceil \frac{R_{i}^{n}\left(l\right)}{LEC}\rceil$$

### 5.2. Building the Interference Patterns

To build the interference patterns we need first to determine the necessary replication level. The Algorithm 1 calculates the necessary replica number for arbitrary scenarios. This algorithm is based on Equation (14), returning vector *RepLevel*, which contains the number of replicas necessary to obtain a probability of non-recovery below *pε* for *i* errors in the SW.
**Algorithm 1.**
*Calc_RepLevel*: Replica Level Calculation **Inputs**: *M*, *LSW*, λ, *pε* **Output**: RepLevel vector**1:**  Determine *C_MAX_* in message set *M* **2:**  Obtain maximum value of *Max_errors* in *P_λ_* (*Max_Errors*; *LSW*) > *pε* **3:  *for*** i = 1 ***to***
*Max_Errors **do*** **4:**    j = 0 **5:    *do***      j = j + 1      P = *P_λ_* (i; *LSW*) × *P_λ_* (1; *C_MAX_*) ^j^ * i    ***while*** (P > *pε*)    *RepLevel*(i) = j  ***end_for*** **6:  *return*** vector *RepLevel*

For illustration purposes, Algorithm 1 was applied to an FTT-CAN system with a 1 Mbps bit-rate, LEC = 2.5 ms, LSW = 1.25 ms, λ = 0.26 errors per second (Aggressive environment, Table 1) and 15 messages with period 5 ms and size 125 bits. The desired global unreliability level *ε_G_* was set to 10^−9^, which translates to *pε* ≈ 10^−16^ by applying Equation (7). Table 2 presents the obtained values. The vector returned by Algorithm 1 for the example above is *RepLevel* = {3, 3, 2, 1}.

Then, we have to compute how many errors must be handled in a single SW and also the maximum number of single errors that have to be accommodated in consecutive SWs, which are the two extreme cases (to see this just calculate the scenario probability by applying Equation(5) to all the considered error scenarios). Algorithm 2 computes these values, termed *max_1cycle* and *max_cycles*, that will be used to build all possible error scenarios.
**Algorithm 2.**
*MaxErrors*: Maximum single consecutive errors and maximum errors in one cycle **Inputs**: LSW, λ, *pε* **Outputs:**
*max_cycles*, *max_1cycle*1:  *max_cycles* = 0, p_error = 1 2:  ***while*** (*p_error* > *pε*) ***do***    *max_cycles* = *max_cycles* + 1    *p_error* = *P_λ_* (1; LSW) ^(*max_cycles*)^  ***end_while*** 3:  *max_cycles* = *max_cycles* − 1 4:  *max_1cycle* = 0, *p_error* = 1 5:  ***while*** (*p_error* > *pε*) ***do***    *max_1cycle* = *max_1cycle* + 1    *p_error* = *P_λ_* (*max_1cycle*; LSW) **  *end_while*** 6:  *max_1cycle* = *max_1cycle* − 1 7:  ***return***
*max_cycles*, *max_1cycle*

The algorithm accepts as inputs *LSW*, λ and *pε*. Lines 1–3 compute the maximum number of consecutive ECs that may be affected by one single error. The reasoning is similar to the one used to derive Equation (14). As in the Poisson process arrivals are independent, the probability of having exactly one error in *n* consecutive cycles is given by the product of the probability of having exactly one error in one cycle, given by *P_λ_* (1; *LSW*). Lines 4–6 compute the maximum number of errors in one SW, being a direct iteration of the Poisson probability function applied to one SW. Table 3 illustrates the results of the algorithm for several scenarios of *LSW* and λ. One can see that *max_cycles* and *max_1cycle* tend to increase with higher values of LSW and λ, as expected. 

**Figure 7:**
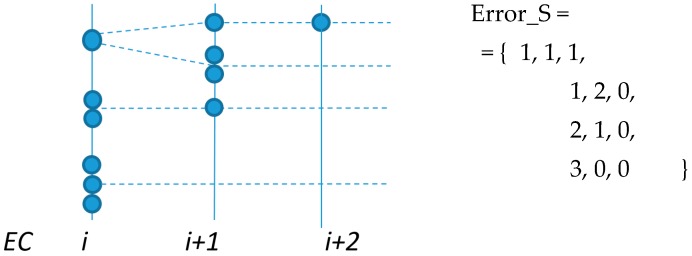
Possible error sequences in consecutive cycles.

After obtaining these two values, we can build the various error sequences or scenarios, *Error_S*, that produce maximum interference. These are the result of all combinations with length *max_cycles* and a maximum of *max_1cycle* errors per EC. For instance, if we consider both *max_cycles* and *max_1cycle* equal to 3, then the possible error combinations are the ones presented in Figure 7. The horizontal-axis in Figure 7 represents the ECs, while errors with probability greater than *pε* are represented by a solid circle. These sequences, when combined with the *RepLevel* vector, allow for us to build the set of *Interf_P* required for computing Equation (16), as explained in the next section.

### 5.3. Server Interference

The error server execution may interfere with any message, having different interference patterns, depending in error sequence and server configuration. We define Indirect Interference on one message when this message does not suffer errors itself, but is delayed by the server executing on behalf of other messages. The Direct Interference corresponds to scenarios where one error affects the message being analyzed. When calculating the response time with direct interference we must also account, at first, with the recovery of *n-1* errors (of indirect interference), in a scenario with *n* errors in total. This aspect is further clarified in Section 5.3.2. The worst-case response time for any message is the maximum of both types of interference. As we will see later on, direct interference is normally more penalizing but it is not always the case, thus the need to compute both scenarios.

#### 5.3.1. Indirect Interference

The server execution may delay the dispatching of lower priority messages, thus causing interference on them. As in this work the server is assigned with the highest priority, to minimize retransmission’s latency, all messages are potentially subject to interference. 

**Figure 8:**
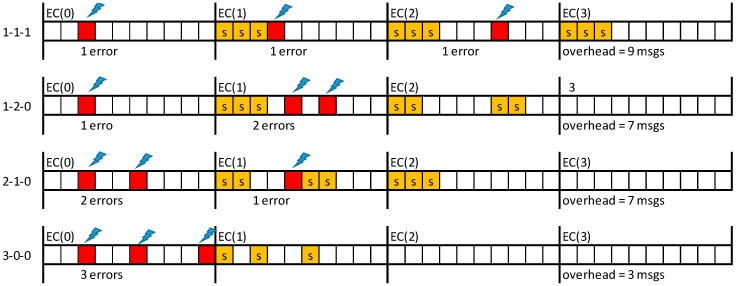
Possible error and recovery scenarios for indirect interference.

Figure 8 illustrates the indirect interference caused by the error scenarios presented in Figure 7. Possible error sequences in consecutive cycles, including all possible error combinations of *max_1cycle* errors that can occur in *max_cycles* cycles, with *RepLevel* = {3, 2, 1}, are presented there. Using a smaller number of errors reduces the server load, and consequently, the indirect interference, thus we just need to consider the combinations depicted in Figure 8.

Algorithm 3 assesses the schedulabity when considering indirect interference on message set *M*, having as inputs *LSW, LEC,* λ, *pε*, and the *RepLevel* vector.

The parameter *Cut_errors* is an auxiliary variable needed to allow for this algorithm to be used both for the Indirect (*Cut_errors* = 0) and Direct (*Cut_errors* = 1) Interference computation. Lines 1–4 determine the *Interf_P* array, required to compute the WCRT of all the messages. Firstly Algorithm 2 bounds the number of errors (per cycle and in consecutive cycles), and then the possible combinations of errors are built. Then the *Error_S* array (the set of error scenarios) is combined with the vector *RepLevel* to obtain *Interf_P*. This vector has size *Max_Patterns*, which corresponds to the number of error scenarios that must be analyzed. Line 5 computes the values that are needed for the non-preemptive blocking free model (Section 3.2). Lines 6–10 apply the extended schedulability test (Equation (16)) to the message set, when considering each one of the error scenarios and interference patterns. If the test fails for any of the patterns, the algorithm returns *Schedulable* = FALSE (Line 10). Otherwise the algorithm returns *Schedulable* = TRUE together with the response time of each message (Line 12), also expressed in number of ECs (Line 11). In fact, the timing granularity of FTT-CAN is the EC duration (LEC) and there is no guarantee on where within an EC a given message will be transmitted.
**Algorithm 3.**
*Ind_Interf*: Response Time with Indirect Server Interference **Inputs**: *M*, *LEC*, *LSW*, *RepLevel*, *Cut_errors,* λ, *pε* **Outputs:**
*Schedulable* (Boolean), *RespTime***1:**  Use **Algorithm 2** to obtain *max_cycles* and *max_1cycle* in LSW **2:**  *max_cycles = max_cycles* − *Cut_errors* **3:**  Build *Error_S* array, considering *max_cycles* and *max_1cycle* **4:**  Build *Interf_P* array by combining the *Error_S* array and *RepLevel* vector;   *Max_Patterns* = number of *Interf_P* lines **5:**  Compute *C_MAX_* and obtain *M^E^*, $C_{MAX}^{E}$ (inflate all transmission times) **6:  *for*** a = 1 ***to***
*Max_Patterns **do*** **7:    *for*** each *m_i_* in *M **do*** **8:**      Compute R_i_ (Equation 16) considering *M^E^* and with *Interf_P(a, j)* × $C_{MAX}^{E}$ and       *Err_S(a, j)* × $C_{error}^{E}$ being added as the cycles progress **9:      *if*** RespTime(i) < R_i_
***then***           RespTime(i) = R_i_         ***end_if*** **10:       *if*** R_i_ > *D_i_*
***then***           ***return***
*Schedulable* = FALSE         ***end_if***       ***end_for***     ***end_for*** **11:**   Transform each *RespTime* vector value from seconds to number of ECs **12**:   ***return***
*Schedulable* = TRUE, *RespTime*

#### 5.3.2. Direct Interference

In this scenario, we consider that the WCRT of a given message occurs when that message suffers the maximum indirect interference from the error server, assigned with highest priority, and one error hits the message itself. To reach this conclusion, just consider the following scenario for an arbitrary message *m_i_*:

1. Once ready, *m_i_* suffers the maximum possible indirect interference (both from high-priority messages and from the error server), being scheduled for transmission in EC *k*;
2. In EC *k*:
  - there are no errors; thus, *m_i_* is transmitted at EC *k*;
  - message(s) other than *m_i_* are affected by errors; thus, *m_i_* is still transmitted in EC *k* (note that errors in EC *k* are handled in EC *k* + 1);
  - *m_i_* is affected by an error; thus, *m_i_* and its replicas are scheduled for the following EC. The WCRT of *m_i_* is then *k* + 1.

Therefore, to assess the schedulability of the message set and obtain the WCRT of the messages considering direct interference, we use the procedure described in Algorithm 4. 

Firstly, we execute the Algorithm 3 with the parameter *Cut_errors* set to 1, because firstly the interference due to indirect errors must be computed with maximum errors minus one, to account for the error directly affecting the message under analysis (Line 1). Then, for each message we assess the impact of the direct error (Lines 3 and 4), and finally we verify if the deadline is violated (Line 5). If the message set is schedulable, the response time of all messages is returned (Line 6).
**Algorithm 4.**
*Direct_Interf*: Response Time considering errors – Direct and Indirect Interference **Inputs**: *M*, *LEC*, *LSW*, *RepLevel* **Output**: *Schedulable* (Boolean), *RespTime_Direct***1:**  Run Algorithm ***Ind_Interf***(*M*, *LEC*, *LSW, RepLevel, Cut_errors* = 1), obtaining *Schedulable* and      *RespTime* **2:  *if***
*Schedulable* == FALSE ***then***    ***return***
*Schedulable* = FALSE  ***end_if*** **3:  *for*** each *m_i_* in *M **do*** **4:**    *RespTime_Direct*(i) = *RespTime(i)* + 1 **5:    *if***
*RespTime_Direct(i)* > D_i_
***then***      ***return***
*Schedulable*=FALSE    ***end_if***  ***end_for*** **6:  *return*** Schedulable=TRUE, *RespTime_Direct*

### 5.4. LSW Optimization

The LSW used in the previous algorithms can be optimized, finding a value that makes the system schedulable, when considering all worst case error scenarios. The optimum LSW is then the minimum value that guarantees that the errors are recovered in the following EC, leaving as much bandwidth as possible to the asynchronous traffic. Algorithm 5 carries out this optimization using a binary search approach. The algorithm has as inputs the message set *M*, *LEC*, the *TM* transmission time (*LTM*), *Guard*, λ, and *Stop_criteria*. *Guard* is a technology-dependent minimum processing time that must be reserved to allow for nodes to decode and process the *TM*. *Stop_criteria*, expressed as a percentage of *LEC*, is the desired precision of the final result and allows for stopping the iterative process. The output is the minimum *LSW* necessary for making the system schedulable.

Firstly, the absolute lower (*LSW_low_*) and upper (*LSW_HIGH_*) LSW bounds are computed. These are, respectively, *C_MAX_* and LEC minus the overheads (*LTM* and *Guard*). Then, in Lines 3-4, the system is tested for feasibility, by giving the maximum time to the LSW. If the test fails, the system is not schedulable. If the system is feasible, the Bisection or Binary Search method is used to find a solution, using as starting point *LSW_low_* and *LSW_HIGH_*. As long as the stop criteria are not met (Line 6), the interval is halved (Line 7) and the schedulability assessed using this value as input, using the algorithms 3 and 4 (Line 8). If this test fails the intermediate point becomes the new lower bound for LSW, otherwise it becomes the upper bound (Lines 10 and 11, resp.). Then, the process is iterated using the new bounds.
**Algorithm 5.**
*Min_LSW*: Obtain Minimum LSW for schedulable system **Inputs**: *M*, *LEC*, *LTM*, *Guard*, λ, *Stop_criteria* **Output**: *LSW***1:**  *LSW_low_* = *C_MAX_*
**2:**  *LSW_HIGH_* = *LEC* – (*LTM* +*Guard*) **3:**  *Sch_high* = Test Schedulability using *LSW_HIGH_* **4:**  ***if** Sch_high* == FALSE ***then*** **5:**    Return -1     ***end_if*** **6:  *while*** (*LSW_HIGH_* − *LSW_low_* > *Stop_criteria* × *LEC*) ***do*** **7:**    *LSW_test_* = (*LSW_HIGH_* + *LSW_low_*)/2 **8:**    *Sch_test* = Test Schedulability using *LSW_test_* **9:    *if***
*Sch_test* == FALSE ***then*** **10:**      *LSW_low_* = *LSW_test_*    ***else*** **11:**      *LSW_HIGH_* = *LSW_test_*    ***end_if***    ***end_while*** **12:  *return***
*LSW_HIGH_*

The complexity of this algorithm is basically the one of the classic Response Time Analysis (RTA) iterative method [27]. Since the RTA is repeated for each error scenario (Indirect and Direct Interference) and iterated for each LSW candidate value, the algorithm execution time is the one of RTA, multiplied by the number of error scenarios, and then multiplied by the number of LSW iterations. The number of error scenarios is equal to 2^*m*−1^ + 2^*m*−2^, when considering *max_cycles* = *max_1cycle* = *m* and seven iterations are need to obtain LSW with 1% error (10 iterations for 0.1% acceptable error). This process is executed off-line, the execution time is acceptable, being for instance less than 1 second for the *Updated_SAE* set. Nevertheless, as this process is computationally intensive, future work will seek to reduce this processing time.

## 6. Results and Discussion

To test the methodology presented in the previous sections we used several benchmarks obtained from the literature. One is the *Updated_SAE* [32], a revised and updated version of the SAE Benchmark [33], which takes into account new modules and functionalities present in 21st century cars. The SAE benchmark is a well-known reference, used in several projects and tools, for instance, in [13,34]. We have also used the *PSA* benchmark [35], and one for an electric prototype vehicle *VEIL* [36]. For the sake of completeness, in Appendix B, the message sets of these three benchmarks are presented.

The *Updated_SAE* message set was used to obtain the individual WCRT of all the messages when considering the Aggressive environment, with λ = 0.26 errors per second. The CAN bit rate is 1 Mbps and LEC was set to 2.5 ms, corresponding to half of the period of the fastest messages, to allow time for their retransmission. If the message set is schedulable for this environment, it is also for the other ones, which have a lower λ, since the interference due to the server execution is lower.

Firstly, we compute LSW using Algorithm 5. The Algorithm 5 triggers the execution of the algorithms described in Section 5 that produce values that are interesting to analyze. For instance, the maximum errors in one cycle (*max_1cycle*) and the maximum consecutive cycles with single errors (*max_cycles*) are both 4. In these conditions, Algorithm 1 returns *RepLevel* = {3, 3, 2, 1}, so the interference pattern array is:

*Interf_P* = { 3, 3, 3, 3; 3, 3, 6, 0; 3, 6, 3, 0; 3, 6, 0, 0; 6, 3, 3, 0; 6, 6, 0, 0; 6, 3, 0, 0; 4, 0, 0, 0 }

With the error server configured according to the description in Section 4.3, with *T_S_* = 1/λ seconds (1538 ECs), C_S_ equal to 12 × 3 × *C_MAX_* and *RepLevel,* as presented in the previous paragraph, the minimum LSW value is equal to 55.1% of the LEC to schedule the message set, including the error server. This algorithm, implemented in MatLab and using a Pentium i7-2670QM computer with 6 GB of memory, took less than 1 second to complete, with 0.1% precision as stopping criterion.

**Figure 9:**
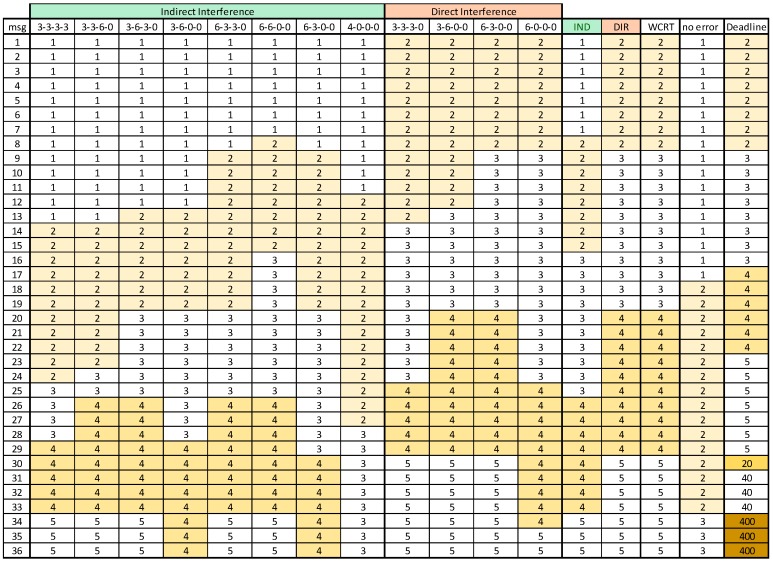
Response time for every message in the *Updated_SAE* set, using each possible interference pattern, when considering Indirect and Direct Interference.

The diverse interference patterns and WCRT for each message are presented in Figure 9, where the “0 errors” column is included for reference, presenting the response times in the absence of errors. 

As expected, the results show a degradation of the WCRT in almost all messages, when compared with the no errors scenario. The first eight messages have a penalty of one EC, since they fit in the EC, in which they become ready, even when the server executes. The messages with lower priorities, e.g., 30 to 36, suffer a stronger response time penalty as they are affected by a higher interference level. 

Figure 9 also confirms that Direct Interference normally dominates Indirect Interference. This is the case in our test scenario. However, even with the same message set but with *RepLevel* equal to {4, 3, 2, 1} there were already cases in which Indirect Interference dominated. This confirms the need to always compute both kinds of interference to determine a safe upper bound to the WCRT.

### 6.1. Assessing the Design Method

**Figure 10:**
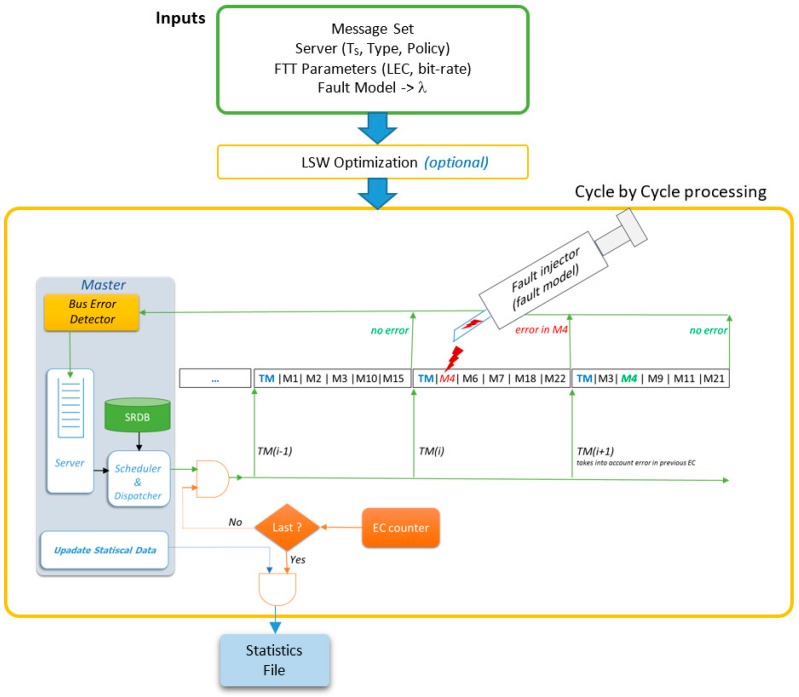
FTT-CAN simulator architecture.

A MatLab simulator of FTT-CAN networks (Figure 10), as previously presented in [12], was extended to include a modified fault injector that follows the fault model described in this paper. The current version of the simulator includes the following new features:

- LSW optimization using binary search;
- server and scheduler functions now include the retransmission replication level; and,
- injector and error detection engine now support multiple errors per EC.

Since the probability of finding two or more errors in consecutive cycles is very low (order of 10^−7^ even in aggressive environments with LSW equal to 2.5 ms), finding such situations in practice or even with simulated execution could take an impractical amount of time. For instance, when considering an aggressive environment, the error scenario 1-1-1-1 will, in average, occur once in every 48.6 thousand years of simulation time. Therefore, our simulator allows not only injecting faults following a Poisson process, but also injecting a full fault pattern chosen randomly from the predefined patterns that correspond to the identified rare scenarios. This allows for observing the impact of such rare random patterns injected in random positions of the message stream.

Using this approach, we simulated three message sets (*Updated_SAE*, *PSA*, and *VEIL*) for 14.4 million cycles of operation, corresponding to 10 h of system operation for the first set and 20 h of operation for the other two sets. This test was repeated 20 times for all three sets without observing any missed deadline, thus with all errors recovered in time, as expected. 

To assess the tightness of our design process, we compared the WCRT generated analytically with the maximum observed response-time in the simulations. Table 4 presents this comparison for the *Updated_SAE* message set, showing that the analytical WCRTs are tight for the messages with higher priority with the potential pessimism growing for the lower priority messages. Nevertheless, note that the values obtained from simulation are not guaranteed to be the absolute maxima, due to the limited simulation time, thus some of the reported differences between computed and observed values may be smaller.

Another measure of the efficiency of our design method is the tightness of the minimum LSW needed to schedule the message sets. Thus, we compared the analytic value obtained from our design approach with the minimum value that we could obtain in simulation, reducing the LSW just until deadline misses started to occur. Table 5 presents these values for the three benchmarks, showing that the analytical minimum value for LSW was roughly between 12% and 16% larger than the one obtained in simulation. Again, note that these differences can be upper bounds to the real differences since there is no guarantee that the simulation captured all of the actual worst-case situations.

Table 5 also shows the minimum LSW needed to schedule the messages sets without errors, showing the impact of error recovery. This impact is particularly large in an Aggressive environment, as considered here, to guarantee timely error recovery. In fact, the minimum LSW that is generated by our approach to account for the Aggressive error scenario is 235%, 208%, and 45% larger than the minimum LSW needed to schedule the corresponding message sets without errors, respectively, *VEIL*, *PSA*, and *Updated_SAE*. One interesting observation is that the relative impact of the error recovery mechanism decreases when the message set bandwidth utilization grows. This is also visible in Table 5.

Nevertheless, we must stress that, thanks to the dynamic scheduling feature, this extra bandwidth configured for the synchronous system is only used when errors do occur. So, the bandwidth effectively used by the recovery mechanism is not determined by the LSW value, but instead by the average features of the error model and corresponds to the error server bandwidth, which in this case is lower than 0.11% of the available bandwidth (Table 5) e.g., the server for the *Updated_SAE* message set has *T_S_* = 1/λ and C_S_ = 12 × 3 × *C_MAX_*.

### 6.2. Comparison with Other Methods

The work in [19] presented another error recovery method for FTT-CAN, based on the native CAN automatic retransmission of messages affected by errors (named *Automatic Retransmission* in Table 6). The mechanism reserves extra time in every SW for the recovery of eventual errors. However, this extra time is left unused in the absence of errors, and is thus less efficient than our proposal (named *Controlled Retransmission* in Table 6). For instance, when considering the fault model presented in this paper and an Aggressive environment, the *Updated_SAE* benchmark message set with the *Automatic Retransmission* mechanism would require space for four retransmissions in every EC. This represents a constant bandwidth of 25.2% (or 12.6% for the other message sets, which have LEC equal to 5 ms). Conversely, our *Controlled Retransmission* mechanism consumes an average bandwidth that is less than the bandwidth of the error server (thirty six maximum messages (3 × 12) in the server period, configured, as described earlier with *T_S_* = 1/λ), which is only 0.108% in this case.

The method presented by Tanasa et al. [15] implies the use of a fixed number of replicas per message period, and is applied in a TDMA fashion (named *Static TT* in Table 6). The number of replicas is found by applying Equation (1), for a defined mission reliability goal. For the three referred benchmarks and with a global reliability of 1–10^−9^, we need to send always four copies of each message (more than 300% overhead accounting for error signaling). The three extra messages sent and their transmission time is always wasted when there are no errors (which is the most common scenario, for the considered BER). This happens because the scheduling is static and needs to cope with every error, without knowing when they will occur. Thus, enough message copies are sent to get a probabilistic assurance that the reliability goal is attained. This is clearly in opposition to our proposal where the bandwidth is only used when needed, i.e., when errors do occur. As shown in Table 6, this method requires a minimum LSW similar to our method for the lightest set (*VEIL*). However, for a set with medium bandwidth utilization (*PSA*), our method already requires a minimum LSW that is 32.4% less than required by Tanasa’s method. For the set with higher utilization (*Updated_SAE*) Tanasa’s method cannot even generate a schedulable solution. Comparing the bandwidth overhead of 15,1% and 29.5% for the two other sets (that corresponds always to more than 300% increase when referenced to each message set utilization bandwidth), with the reserved bandwidth of less than 0.11% used by our method is even more revealing.

Further, using the results available in the statistic files of the performed simulations (view Section 6.1), the bandwidth that was used in the recovery process was also calculated. The average value found was very low, being 0.00047%, 0.00093%, and 0.0025% of the available bandwidth for the *VEIL, PSA,* and *Updated_SAE* benchmarks, respectively. These values are much lower than the bandwidth configuration server values of 0.105% and 0.108%, as the full server capacity was never used.

Nevertheless, our approach consists of detecting errors by the end of the EC and scheduling retransmissions in the following EC, which leads to an error recovery latency of one EC. Conversely, both the other methods allow error recovery in the EC in which they occur, thus being faster.

### 6.3. LSW Optimization with Random Sets and BW Required by the Error Recovery Mechanism

**Figure 11:**
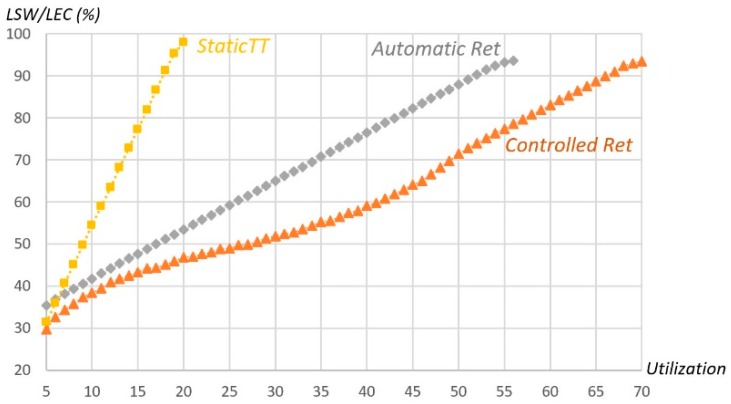
Average requirement for minimum LSW vs message set bandwidth utilization.

Multiple runs of the LSW optimization module were carried out to assess the performance of the three methods referred in the previous section, concerning the minimum LSW value required to attain the desired error responsivity, as a function of the message set utilization. The bandwidth utilization range varied between 5% and 70%, in steps of 1%. Message payload length varied from 1 to 8 bytes and message periods from 2 to 15 ECs, with implicit deadline (i.e., equal to period), both with uniform distribution. LEC was set to 2.5 ms and one thousand message sets were generated for each utilization value. The results are presented in Figure 11, averaging the obtained minimum LSW of the 1000 sets of each utilization point.

*Static TT* shows a better performance than *Automatic Retransmission* (smaller minimum required LSW) but only for low utilization values (less than 8%). The *Controlled Retransmission* method always shows better performance than all of the other ones. When compared to *Automatic Retransmission*, it is slightly better for sets with utilization lower than 15% and grows for larger utilizations, requiring up to about 16% less in the LSW parameter. The picture also shows that our method allows for attaining much higher utilization levels than the competing ones, for the same reliability goal, allowing up to 70% utilization against 20% and 55% from *Static TT* and *Automatic Retransmission*, respectively.

**Figure 12:**
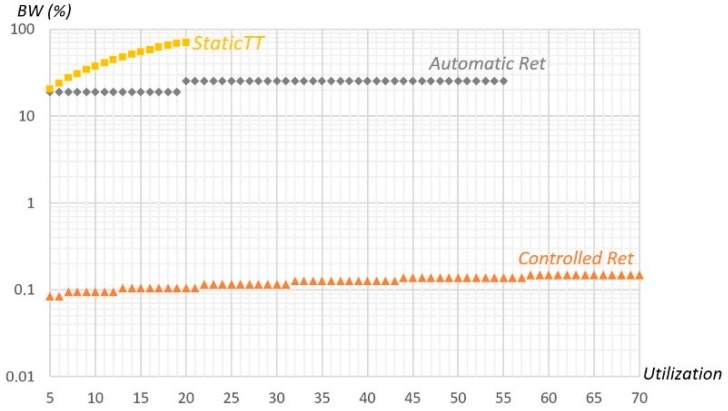
Bandwidth required by each method (with LEC = 2.5 ms and λ = 0.26).

Figure 12 represents the bandwidth required by the different error recovery mechanisms as a function of the message set utilization, being obtained with random message sets using LEC equal to 2.5 ms, an Aggressive environment, and the other parameters as in the previous experiences. In *Static TT*, the required bandwidth is proportional to the number of copies needed for each message, also including error signaling, being always more than 300% of the corresponding message utilization bandwidth, as four copies per message are required to attain the desired reliability level. Thus, more than 70% of the total bandwidth is allocated to the recovery mechanism, even for message sets with a utilization of only 20%. *Automatic Retransmission* reserves slack time in each EC to recover the maximum number of errors considered (equal to *max_1cycle*), which in the studied cases is 3 or 4, corresponding to 19.0% or 25.2% of the available bandwidth. Therefore, the overhead is essentially constant, having a step in the utilization transition from 19% to 20%, corresponding to the situation in which the errors that need to be handled changes from 3 to 4 per EC. As for the *Controlled Retransmission* mechanism, the reserved bandwidth is the one of the error server. The server configuration depends on the fault arrival rate and maximum *RepLevel* configuration value, and using *T_S_* equal to 1/λ, the necessary capacity varies between 8 × 3 × *C_MAX_* and 14 × 3 × *C_MAX_*, which is only 0.084% and 0.147% of the 1 Mbps available bandwidth. This figure clearly shows the superiority of our method, since its required bandwidth is at least two orders of magnitude inferior to the other methods.

## 7. Implementation Issues and Extension

In our proposal, the achievement of high reliability imposes that the Master node must be capable of detecting all bus errors to reschedule messages affected by errors. Due to the error signaling mechanisms of CAN, this is true most of the times, but can fail in rare scenarios, causing Inconsistent Message Omission, as described by Rufino [37], where some nodes receive correctly a message, while others do not. As in the synchronous window the native CAN’s automatic retransmission mechanism is disabled, the probability of encountering this scenario becomes significantly higher [19], thus decreasing the global reliability level.

To solve this problem, a possible solution is to scan individually the last bits of the CAN frame (last bit of EOF and following three bits of the IFS field) and, if a dominant value is read there, the master node considers that an error, changing this way the CAN rule in interpreting these bit values. This new error detection mechanism can only fail if four errors hit these last four bits. When considering single bit errors, even in an aggressive environment, this scenario has a probability much lower than the 10^−9^ incidents per hour ultra-reliability number. This feature is relatively simple to implement using, for example, a small FPGA and it would have to reside in the Master node, only.

In the proposed mechanism, when an error occurs, the Master needs to request the transmission of multiple replicas. However, the protocol defines that the *TM* message encodes the transmission request of each message in only one bit (see Figure 1) and due to the limited payload of CAN messages (maximum of 8 bytes) there is not enough space in the *TM* to add information on the number of replicas to transmit. Thus, the FTT-CAN protocol must be adapted to deal with this situation. We propose adding another message, called *TM_Ret*, that is transmitted only in ECs in which there are replicated messages. The *TM_Ret* payload is coded as follows:

- 1 byte per message to retransmit; and,
- each byte is composed of two segments—2 bits to encode the number of replicas (00, 01, 10, 11 for two, three, four, or five replicas, respectively) and 6 bits to encode the message ID.

The use of *TM_Ret* with the referred transmission rules and coding scheme minimizes the extra traffic transmitted by the Master. This mechanism also has the advantage of being fully compatible with legacy nodes that do not transmit critical messages. To take advantage of the proposed error recovery mechanism, slave nodes have to adjust the FTT-CAN stack to receive and interpret the *TM_Ret* and act accordingly. Also, this message has an impact on the maximum LSW available and on the timeliness of aperiodic messages.

Finally, our mechanism is also compatible with the recent CAN-FD [7] protocol that is increasingly gaining acceptance. In this case, since the payload can be longer, up to 64 bytes, the referred *TM_Ret* is not needed and its information can be transmitted in additional bytes of a normal TM message, being this is a more bandwidth efficient approach.

## 8. Conclusions

In any real network, transmission errors are unavoidable and thus, a method to guarantee the delivery of messages, despite error occurrence, is necessary. This is especially true if a high reliability system is needed. In this paper, we presented a methodology to recover 1-bit errors in a simplex Controller Area Network (CAN or CAN-FD) running a time-triggered protocol. We showed that using on-line traffic scheduling plus a specifically designed server to handle retransmissions allows very large error recovery bandwidth savings while maintaining a high reactivity to errors, when compared with other error recovery approaches for time-triggered communication on CAN available in the literature. Conversely, the properties of our mechanism are closer to those of event-triggered CAN. However, the real-time guarantees in this case depend completely on the accuracy of the error model since the retransmissions are normally uncontrolled. Our solution explicitly controls retransmissions using a server, thus enforcing the properties of the considered error model. This is attained by including a run-time module that checks the arriving error patterns and limits the server execution to the patterns used in its configuration (see Section 5.2). Thus, the impact on the system timeliness of errors occurring beyond the model is strictly bounded.

To implement our mechanism we used the FTT-CAN protocol, taking advantage of its on-line scheduling of the time-triggered traffic. We provided a detailed description of the proposed method, including the considered fault model, choice of server, and its parameterization and clear problem definition. We also identified the most demanding error scenarios and determined the level of replication needed to guarantee error recovery in the next FTT-CAN cycle. All of the methods were discussed in detail, including the corresponding algorithms.

We tested the proposed error recovery mechanism simulating several well-known benchmarks, as well as random message sets and we compared it with other methods available in the literature. We showed that the proposed method is effective in recovering errors in time-triggered messages using approximately two orders of magnitude less average bandwidth than other approaches, while the instantaneous bandwidth required by our method (average of the random sets) is also always lower than the one used by other methods, allowing for the application of the error recovery method to message sets with greater bandwidth utilization. On the other hand, our method can only recover errors in the following protocol cycle while other approaches can recover in the cycle in which the error occurred. This may force our mechanism to use shorter cycles to handle fast messages appropriately.

Currently, we are working to extend our approach to other than the Poisson single bit error model, so that we can accurately apply our method to situations with error bursts and periods of sustained higher interference. The optimization and reduction of the complexity in the schedulability and analysis techniques is another direction to pursue as future work.

## Figures and Tables

**Table 1:** Bit Error Rate (BER) measurements in Controller Area Network (CAN) [18].

Environment	BER
Benign	3.0 × 10^−11^
Normal	3.1 × 10^−9^
Aggressive	2.6 × 10^−7^

**Table 2:** Number of replicas needed for a target reliability level in an Aggressive environment.

Scenario	Replica Number (n msgs)	p_fail_	*ε_G_* = 10^−9^	Overhead (Number msgs)
1 error, triple ret	3	1.12 × 10^−17^	OK	3
2 errors, triple ret	3	3.62 × 10^−21^	OK	6
3 errors, double ret	2	1.81 × 10^−21^	OK	6
4 errors, simple ret	1	6.04 × 10^−21^	OK	4

**Table 3:** Maximum consecutive cycles (max_cycles) with single error and maximum number of errors in one cycle (max_1cycle), for various values of LSW and λ, using *pε* = 10^−16^.

LSW(ms); λ	max_cycles	max_1cycle
2.5; 0.026	3	3
2.5; 0.26	5	4
25; 0.026	5	4
25; 0.26	7	6

**Table 4:** Comparing analytical WCRT with that observed in simulation for the *Updated_SAE* message set with LSW = 55.1% of LEC, considering an Aggressive environment.

ID	Design	Simul	ID	Design	Simul	ID	Design	Simul	ID	Design	Simul
1	2	2	10	3	2	19	3	3	28	4	4
2	2	2	11	3	2	20	4	3	29	4	4
3	2	2	12	3	2	21	4	4	30	5	4
4	2	2	13	3	3	22	4	3	31	5	4
5	2	2	14	3	3	23	4	3	32	5	4
6	2	2	15	3	3	24	4	3	33	5	4
7	2	2	16	3	3	25	4	3	34	5	4
8	2	2	17	3	3	26	4	4	35	5	4
9	3	2	18	3	3	27	4	3	36	5	4

**Table 5:** Minimum LSW configuration value by design and simulation.

Message Set	VEIL	PSA	Updated_SAE
LEC (ms)	5	5	2.5
RepLevel	3-2-2-1	3-3-2-1	3-3-2-1
Bandwidth utilization (@1Mbit/s)	4.4%	9.1%	27.9%
Error server bandwidth(configuration)	0.105%	0.105%	0.108%
LSW/LEC without errors (by design)	7.1%	11.9%	37.9%
LSW/LEC with errors+server (by design)	23.8%	28.0%	55.1%
LSW/LEC with errors+server (simulation)	21.1%	24.8%	48.4%
Pessimism (design over simulation)	12.8%	12.7%	13.9%

**Table 6:** Comparison of minimum LSW and bandwidth (BW) requirement with different design methods.

	Minimum LSW			Configuration BW		
**Message set**	VEIL	PSA	Updated_SAE	VEIL	PSA	Updated_SAE
Controlled Retransmission	23.8%	28.0%	**55.1%**	**0.105%**	**0.105%**	**0.108%**
Automatic Retransmission	**19.8%**	**24.5%**	60.0%	12.6%	12.6%	25.2%
Static TT	22.3%	41.4%	X	15.1%	29.5%	X

## Appendix A. Acronyms

**Table:**

**Symbol**	**Meaning**	**Units**
AW	Asynchronous Window	
BER	Bit-Error Rate	
EC	Elementary Cycle	
Err_S	Array with error scenarios	
FTT	Flexible Time-Triggered	
GP	Global success Probability	
Interf_P	Array with interference pattern	
λ	Process intensity	fauts/second
LEC	Length of Elementary Cycle	seconds
LTM	Length of Trigger Message	seconds
LSW	Length of Synchronous Window	seconds
max_cycles	Maximum number of consecutive ECs with 1 error per EC	
max_1cycle	Maximum number of errors in one EC	
MT	Mission Time	hours/seconds
*pε_i_*	Acceptable failure probability in message *i*, in one invocation, obtained by Equation (7)	
*P_λ_* (*k*; τ)	Probability of obtaining *k* errors in τ seconds, with process intensity λ	
RepLevel	Vector with replica number (function of number of errors)	
R_i_	Worst case response time of message i	seconds or number of ECs
SW	Synchronous Window	
TM	Trigger Message	
*T_S_*	Error server period	seconds
WCRT	Worst Case Response Time	seconds

## Appendix B. Benchmarks Message Sets

The message sets referred in Section 6 have the characteristics presented in the 3 following tables. There, ID is the CAN identifier (lower ID means higher priority), T is the period in ms, T_LEC_ the corresponding value in number of LECs and DLC is the payload length in bytes.

**Table B1:** *Updated_SAE* message set.

ID	T/T_LEC_	DLC	ID	T/T_LEC_	DLC	ID	T/T_LEC_	DLC	ID	T/T_LEC_	DLC
1	50/20	1	10	7.5/3	1	19	10/4	6	28	12.5/5	5
2	5/2	2	11	7.5/3	1	20	10/4	2	29	12.5/5	3
3	5/2	1	12	7.5/3	1	21	10/4	3	30	50/25	1
4	5/2	2	13	7.5/3	1	22	10/4	2	31	100/40	4
5	5/2	1	14	7.5/3	4	23	12.5/5	2	32	100/40	1
6	5/2	2	15	7.5/3	4	24	12.5/5	2	33	100/40	1
7	5/2	1	16	7.5/3	4	25	12.5/5	2	34	1000/400	3
8	5/2	1	17	10/4	1	26	12.5/5	2	35	1000/400	1
9	7.5/3	1	18	10/4	2	27	12.5/5	4	36	1000/400	1

FTT-CAN configuration parameters—LEC = 2.5 ms; bit-rate = 1000 Kbps, all deadlines equal to periods except D_1_ = 5 ms, D_30_ = 20 ms.

**Table B2:** *PSA* message set.

ID	T/T_LEC_	DLC	ID	T/T_LEC_	DLC	ID	T/T_LEC_	DLC	ID	T/T_LEC_	DLC
1	10/2	3	7	20/4	3	13	50/10	8	19	100/20	7
2	10/2	5	8	20/4	4	14	50/10	8	20	100/20	7
3	10/2	5	9	20/4	5	15	50/10	8	21	150/30	2
4	10/2	8	10	40/8	5	16	100/20	1	22	150/30	4
5	15/3	2	11	50/10	5	17	100/20	6	23	200/40	4
6	15/3	4	12	50/10	5	18	100/20	7			

FTT-CAN configuration parameters—LEC = 5 ms; bit-rate = 1000 kbps; all deadlines equal to periods.

**Table B3:** *VEIL* message set.

ID	T/T_LEC_	DLC	ID	T/T_LEC_	DLC	ID	T/T_LEC_	DLC	ID	T/T_LEC_	DLC
1	10/2	2	6	20/4	4	11	100/20	8	16	1000/200	1
2	10/2	2	7	50/10	2	12	250/50	1	17	1000/200	2
3	10/2	4	8	50/10	3	13	500/100	2	18	1000/200	8
4	20/4	1	9	100/20	4	14	500/100	2	19	1000/200	8
5	20/4	2	10	100/20	8	15	1000/200	1			

FTT-CAN configuration parameters—LEC = 5 ms; bit-rate = 1000 kbps; all deadlines equal to periods.
